# Structural basis of receptor-binding adaptation of human-infecting H3N8 influenza A virus

**DOI:** 10.1128/jvi.01065-24

**Published:** 2025-02-24

**Authors:** Tianjiao Hao, Yufeng Xie, Yan Chai, Wei Zhang, Di Zhang, Jianxun Qi, Yi Shi, Hao Song, George F. Gao

**Affiliations:** 1 Beijing Life Science Academy 679188 , Beijing, China; 2 Department of Basic Medical Sciences, School of Medicine, Tsinghua University 593742 , Beijing, China; 3 CAS Key Laboratory of Pathogen Microbiology and Immunology, Institute of Microbiology, Chinese Academy of Sciences (CAS) 653911 , Beijing, China; 4 Faculty of Health Sciences, University of Macau 546496 , Macau SAR, China; 5 Beijing Key Laboratory of Emerging Infectious Diseases, Institute of Infectious Diseases, Beijing Ditan Hospital, Capital Medical University 12638 , Beijing, China; 6 Beijing Institute of Infectious Diseases, Beijing, China; 7 National Center for Infectious Diseases, Beijing Ditan Hospital, Capital Medical University 12638 , Beijing, China; University Medical Center Freiburg, Freiburg, Germany

**Keywords:** avian influenza virus, H3N8, human infecting, dual receptor binding, structure, host jump

## Abstract

Recent avian-origin H3N8 influenza A virus (IAV) that have infected humans pose a potential public health concern. Alterations in the viral surface glycoprotein, hemagglutinin (HA), are typically required for IAVs to cross the species barrier for adaptation to a new host, but whether H3N8 has adapted to infect humans remains elusive. The observation of a degenerative codon in position 228 of HA in human H3N8 A/Henan/4-10/2022 protein sequence, which could be residue G or S, suggests a dynamic viral adaptation for human infection. Previously, we found this human-isolated virus has shown the ability to transmit between ferrets via respiratory droplets, with the HA-G228S substitution mutation emerging as a critical determinant for the airborne transmission of the virus in ferrets. Here, we investigated the receptor-binding properties of these two H3N8 HAs. Our results showed H3N8 HAs have dual receptor-binding properties with a preference for avian receptor binding, and G228S slightly increased binding to human receptors. Cryo-electron microscopy structures of the two H3N8 HAs with avian and human receptor analogs revealed the basis for dual receptor binding. Mutagenesis studies reveal that the Q226L mutation shifts H3N8 HA’s receptor preference from avian to human, while the G228S substitution enhances binding to both receptor types. H3N8 exhibits distinct antigenic sites compared to H3N2, prompting concerns regarding vaccine efficacy. These findings suggest that the current H3N8 human isolates are yet to adapt for efficient human-to-human transmission and further continuous surveillance should be implemented.

**IMPORTANCE:**

Influenza virus transmission remains a public health concern currently. H3N8 subtype influenza A viruses infect humans and their HAs acquire the ability to bind to both human and avian receptors, posing a threat to human health. We have solved and analyzed the structural basis of dual receptor binding of recently human-infecting H3N8 HA, and we demonstrate that the G228S enhances human receptor binding and adaptation. We also found that HN/4-10 H3N8 HA has distinct antigenic sites, which challenges vaccine efficacy. Taken together, our work is critical to the prevention and control of human H3 influenza virus infection.

## INTRODUCTION

Influenza A virus (IAV) is a negative-stranded, segmented RNA virus belonging to the family *Orthomyxoviridae* and a major cause of pandemic and seasonal influenza in humans (1, 2). In wild birds, IAVs are classified into 16 subtypes by the surface glycoprotein hemagglutinin (HA) and 9 subtypes by neuraminidase (NA), the 2 most antigenically variable viral surface glycoproteins (3, 4). The discovery of IAV-like viruses H17N10 and H18N11 in bats has further extended the host range of IAVs (4, 5), while the uncharacterized H19 subtype highlights the evolutionary diversity of IAV (6, 7).

Cross-species transmission can significantly increase the potential for IAVs to cause human pandemics, raising major concerns (8). One of the key factors for host jump is the switch in sialic acid receptor-binding specificity of the virus, mostly determined by amino acid residue substitutions that occurred in the receptor-binding site (RBS) of glycoprotein HA (9). Among avian influenza viruses (AIVs) that have jumped from animals to humans [H7N9, H5N1, H9N2, and H10N8 (10–12)], H7N9 viruses isolated in Shanghai in 2013 had typical avian receptor-binding capacity and were isolated in only a few cases, whereas H7N9 viruses isolated in Anhui with residue substitutions (S138A/G186V/T221P/Q226L, H3 numbering) had dual human and avian receptor-binding capacity (11) and became the primary agents of the subsequent H7N9 influenza epidemic that caused more than 615 deaths (http://www.who.int/en) (13). Based on the results of structural studies, G186V and Q226L have been identified as key sites for dual receptor binding (11, 13). These substitutions in the RBS are crucial in altering the receptor binding preferences of IAVs and require considerable attention and ongoing monitoring.

The H3Ny IAVs have a broad host range (14, 15), yet only H3N2 is generally capable of infecting humans and spreading within the human population and has become a major contributor to seasonal influenza. In addition to increased N-glycosylation sites and altered surface antigen sites (16), substitutions in their RBS also play an important role in the circulation of H3N2 (9, 17). The G228S and Q226L substitutions conferred A/Aichi/2/1968 (1968 H3N2) the ability to bind human receptor (18). With evolution, in A/Hong Kong/4443/2005 (2005 H3N2) strain, residues N225, F193, I226 in the RBS affected the binding affinity of HA for human receptor (19). Studies on other H3 variants for human infection, however, are yet lacking.

H3N8 AIVs typically spread through wild birds, but they have already crossed the barrier to infect mammalian species, including dogs, seals, and horses (20–25). Previous research has also shown that H3N8 AIVs can infect pigs by observing lung lesions (26). However, H3N8 AIVs have never been reported to infect humans before until April, May 2022 and March 2023. Three cases of H3N8 IAV infection in two young children and a 56-year-old woman were reported in Henan, Hunan, and Guangdong provinces of China (27–29), which resulted in the death of the 56-year-old woman (30). The first case of the H3N8 virus infecting humans in Henan caused severe symptoms in the patient, including fever, nasal discharge, and sneezing and eventually progressed to severe acute respiratory distress (27). Besides, the other two strains of H3N8 that infected humans had the same origin and were very similar to the avian influenza epidemic strain in Guangdong in 2021 (31). Epidemiological research suggests that the novel human-infecting H3N8 virus is a spillover from chickens, and phylogenetic analysis showed that the H3N8 viruses were evolving as a triple reassortment event with the Eurasian avian H3 gene, the North American avian N8 gene, and the G57 genotype H9N2 internal genes, suggesting that the virus is avian originated and is typical of cross-species transmission (31). The discovery of a degenerative codon at position 228 in the human H3N8 A/Henan/4-10/2022 HA protein sequence, which may encode an amino acid residue G or S, suggests dynamic viral adaptation for human infection. Chicken-derived or human H3N8 IAVs can replicate in human bronchial tissues and mice (32, 33) and undergo a G228 to S228 mutation in ferret transmission experiments (34). Our recent studies have demonstrated that the human-isolated virus has the capacity to transmit between ferrets via respiratory droplets, with the HA-G228S and PB2-E627K substitution mutations identified as pivotal factors in facilitating airborne transmission among ferrets (35). It is, therefore, highly likely that the evolution of H3N8 strains will lead to further infections in humans. However, the receptor-binding specificity of the HA protein from this human-infecting H3N8 IAV and the structural basis for its ability to infect humans remain unknown.

Here, we generate the soluble HA protein of A/Henan/4-10/2022 H3N8 (HN/4-10 H3N8) to evaluate the receptor-binding properties to human receptor glycans α2-6 and avian receptor glycans α2-3 at the molecular level by surface plasmon resonance (SPR) and tissue staining. We find that the human-infecting H3N8 HA has a dual receptor-binding property with a preference for binding to avian receptor, and the G228S substitution slightly increased binding to human receptor. We then solve the structures of the H3N8 HA protein in complex with the human receptor or the avian receptor, and identified the structural basis of H3N8 infection in humans. These results can help to understand the molecular mechanism of H3N8 cross-species infection in humans, to assess the possibility of its further transmission and the evolutionary trend of IAVs, which can play a key role in scientific prevention and early-warning of an influenza pandemic.

## RESULTS

### Evolutionary and key amino acid residue analyses in the HA RBS of H3Ny viruses

**Fig 1:**
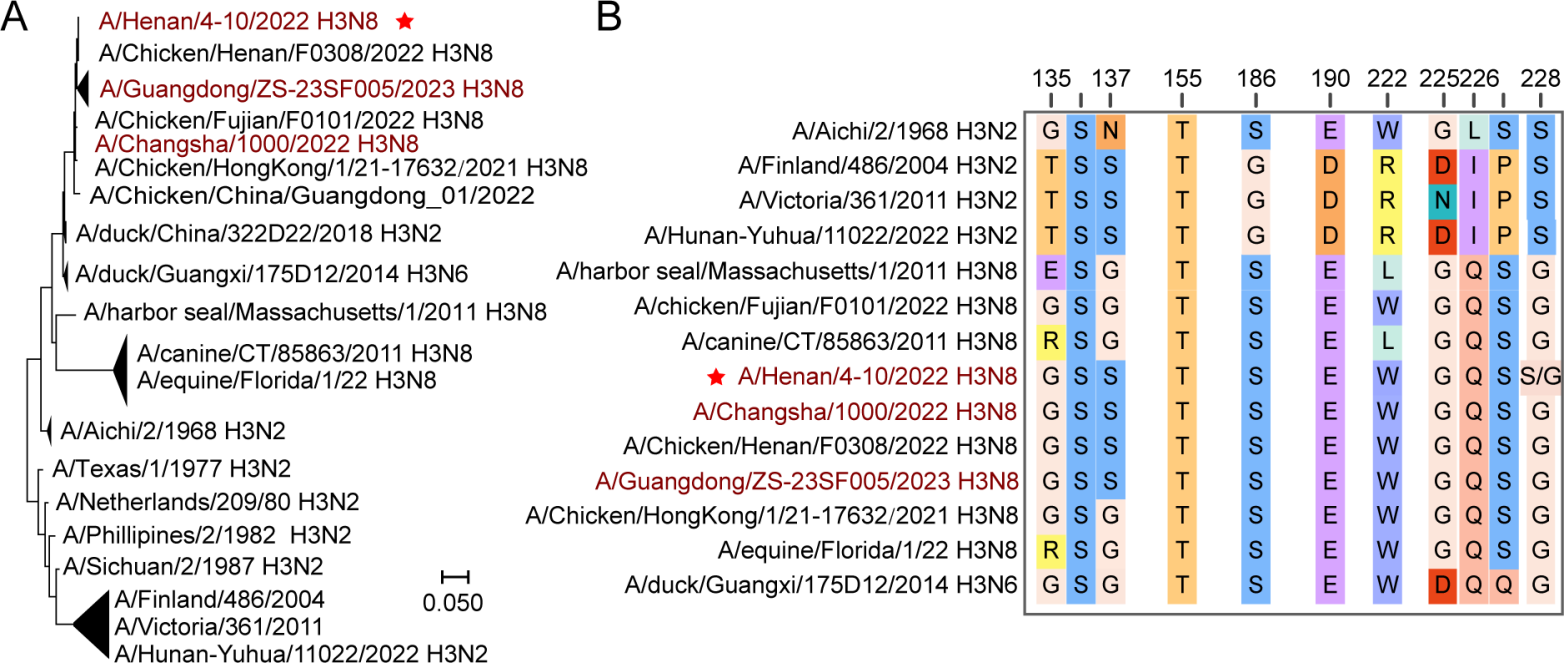
Evolutionary analysis of the H3 subtype HA and comparison of key amino acids in RBS. (**A**) Phylogenetic tree of the representative HAs from different H3 subtype strains. (**B**) Alignment of the key amino acids of HN/4-10 H3N8 HA RBS with other H3s. Human infecting H3N8 strains were colored dark red.

Phylogenetic analysis revealed that the HN/4-10 H3N8 HA still fell within avian H3 subtype, but the HN/4-10 H3N8 HA was evolutionarily distant from the seasonal flu human H3N2 HA lineage even though both are capable of causing human infections (Fig. 1A). Compared to current H3N2 strains, the 1968 H3N2 HA is more closer to avian H3N8 HA, indicating that the evolution of H3 from avian and human origins took separate paths. We also compared the amino acid sequence of the RBS of HN/4-10 H3N8 HA with that of human, chicken, duck, dog, seal, and horse H3 strains (Fig. 1B). For the amino acid (AA) at site 135, HN/4-10 H3N8 is conserved among the same origin avian H3N8 and A/Aichi/2/1968 (1968 H3N2) strains but differs from those of canine and equine H3N8. Surprisingly, the AA 137 residue is mutated into Ser in HN/4-10 H3N8 strain and the strains nearby, unlikely H3N8 of other species, but the same to A/Finland/486/2004 (2004 H3N2), A/Victoria/361/2011 (2011 H3N2) and A/Hunan-Yuhua/11022/2022 (2022 H3N2). Previous research has shown that AA substitutions at sites 186, 190, 225, 226, and 228 (H3 numbering) are significant for receptor-binding adaptations in a variety of HA subtypes, including H1, H2, H3, H4, H5, H6, and H7 (9). AA 186 and AA 190 sites are conserved in all H3N8 and 1968 H3N2. As for the 222 and 225 sites, which may affect the sialic acid-binding affinity in 2004 H3N2 (19), HN/4-10 H3N8 is partial conserved as Trp and Gly as other H3N8. L226 and S228 are often considered signatures for H2 and H3 AIV that change receptor-binding preference from avian receptor to human receptor (9). The presence of Q226 in HN/4-10 H3N8 indicates that the virus may be still a typical AIV strain. However, the AA 228 location is also crucial for binding to human receptors. A sequence degeneracy in the HN/4-10 H3N8 HA exists at position AA 228 and can be translated to either serine or glycine. Notably, glycine is present in AA 228 of all other H3N8 strains. This switch from G to S could be critical for this virus to adapt to human infection.

### Human-origin H3N8 HAs have dual receptor-binding properties

**Fig 2:**
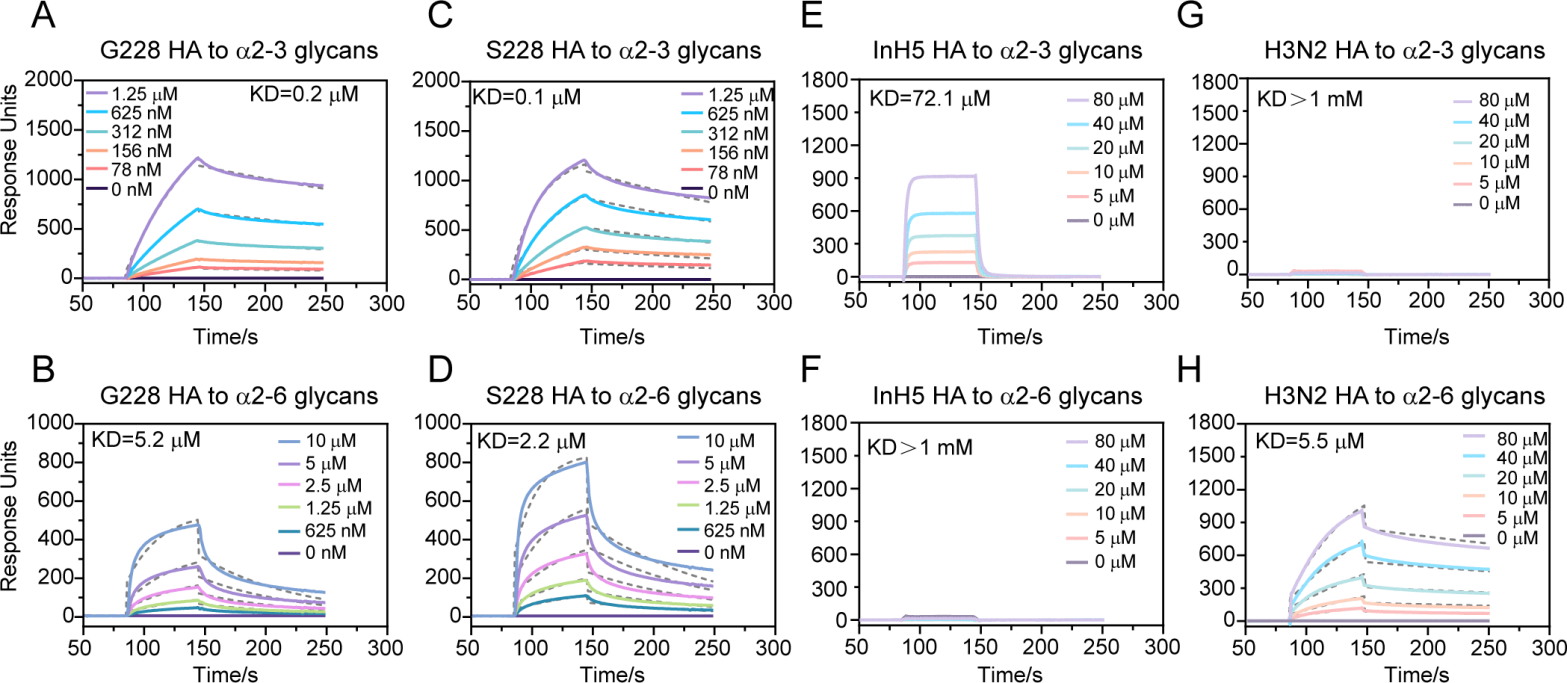
Receptor-binding properties of human-origin HN/4-10 H3N8 G228 and S228 HA. (**A and B**) BIAcore diagrams of G228 binding to the α2-3-linked and α2-6-linked sialylglycan receptor. (**C and D**) BIAcore diagrams of S228 binding to the α2-3-linked and α2-6-linked sialylglycan receptor. The glycans are immobilized on the chip and the protein concentration is shown. (**E and F**) InH5 HA and (**G and H**) 2017 H3N2 HA are positive controls for α2-3 and α2-6 glycans binding, respectively. Response units were plotted against protein concentration. The plots and K_D_ values are one representative data set from three independent experiments. Dashed lines represent the data fitted by BIAevaluation software 4.1 using a 1:1 Langmuir binding model.

To investigate the receptor-binding specificity of the HN/4-10 H3N8 HA protein, we expressed two soluble recombinant HAs with G or S at position 228 (H3N8 G228, S228 HAs) (Fig. S1A and B) and examined their affinities to α2-3 glycans (avian receptor) and α2-6 glycans (human receptor) by surface plasmon resonance (SPR). The results showed that H3N8 HAs have dual receptor-binding properties (Fig. 2). Both G228 and S228 HAs have strong affinities for α2-3 (0. 2 µM/0.1 µM) as expected (Fig. 2A and C). For human receptor binding, both recombinant proteins G228 and S228 had the ability to bind α2-6 glycans, and the affinity of S228 (2. 2 µM) was slightly higher than that of G228 ( 5.2 µM) (Fig. 2B and D).

As controls, HA from A/Indonesia/5/2005 (InH5) is shown to specifically bind the avian receptor (36) (Fig. 2E and F) and A/Kansas/14/2017 (2017 H3N2) HA is shown to specifically bind the human receptor (Fig. 2G and H).

To evaluate the effect of the mutation at position 228 on the HN/4-10 H3N8 HA protein, we also investigated the thermostability of both G228 and S228 proteins. Temperature-dependent circular dichroism (CD) identification showed that the midpoint transition temperatures (Tm) of S228 and G228 were not significantly different (45.4°C and 45.2°C) (Fig. S1C), indicating that the HA stability was not affected by this substitution.

### Human-origin H3N8 HAs bind to human trachea and duck small intestine efficiently

**Fig 3:**
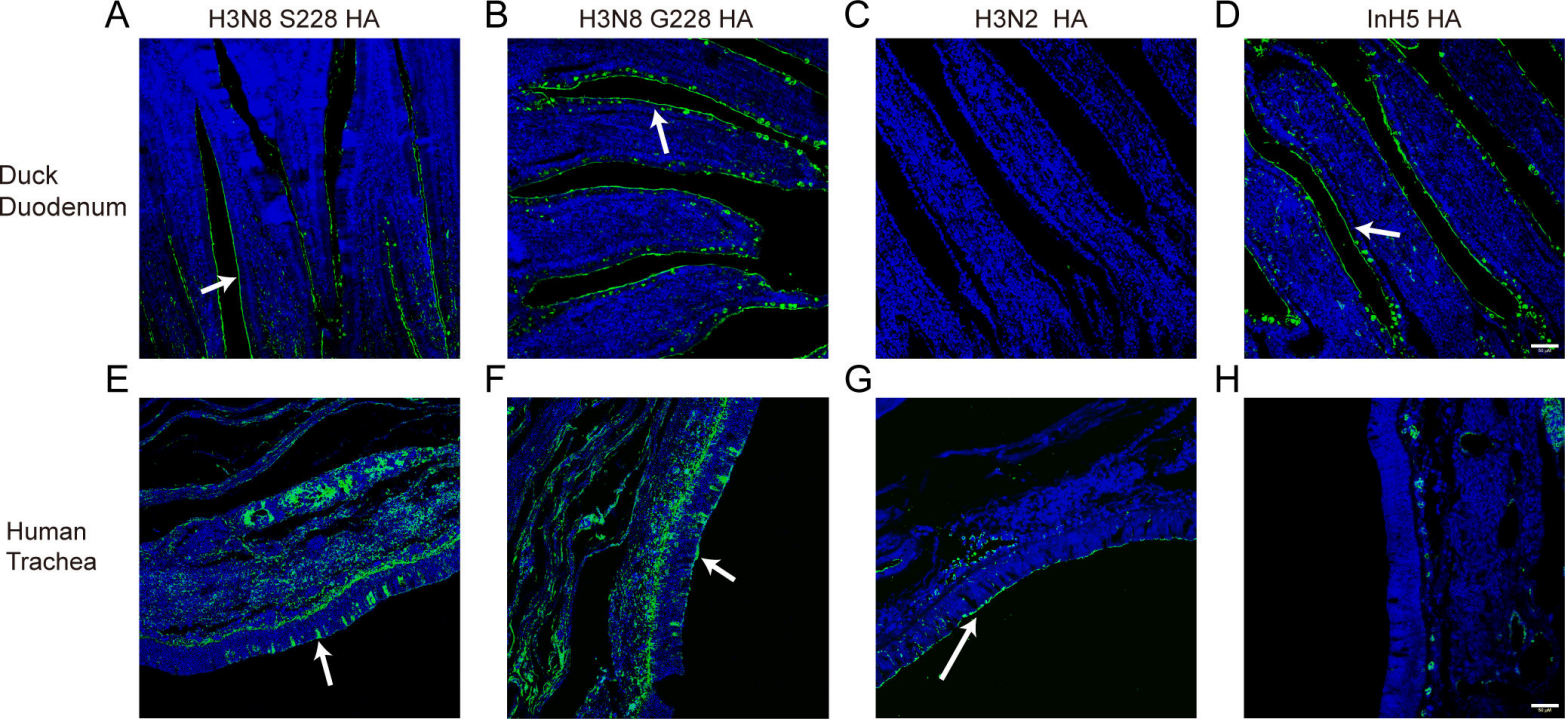
Staining of human trachea and duck small intestine with HN/4-10 H3N8 HAs and other HAs. (**A–D**) HN/4-10 H3N8 S228, G228, 2017 H3N2, InH5 HAs staining duck duodenum tissue sections. (**E–H**) HN/4-10 H3N8 S228, G228, 2017 H3N2, InH5 HAs staining human trachea tissue sections. Specific staining areas are green and indicated by white arrows. Scale bar: 50 µm.

To further assess the host tissue tropism of HN/4-10 H3N8 HA to α2-3 and α2-6 terminated glycans, we stained tissue sections of human trachea and duck small intestine with soluble recombinant HA S228 and G228 (Fig. 3). The apical surface of human trachea shows mainly different glycan receptors terminated by α2-6 sialic acid linkage, whereas the apical surface of duck intestine is covered with different glycan receptors terminated by α2-3 sialic acid linkage (17). In human trachea sections, both S228 and G228 HAs were able to generate specific fluorescences on the surface of tissue cilia similar to the 2017 H3N2 HA. Similarly, when staining duck intestinal tissue, H3N8 S228 and G228 HAs significantly stained the surface of duck intestinal cilia, comparable to InH5 HA, which has a high affinity for α2-3 glycans (37). Thus, for HN/4-10 H3N8 viruses, the binding capacity of human receptor α2-6 glycans can be obtained regardless of whether the 228 position is G or S, indicating that HN/4-10 H3N8 HA has acquired the ability to bind dual receptors.

### Structural basis of HN/4-10 H3N8 receptor-binding

**Fig 4:**
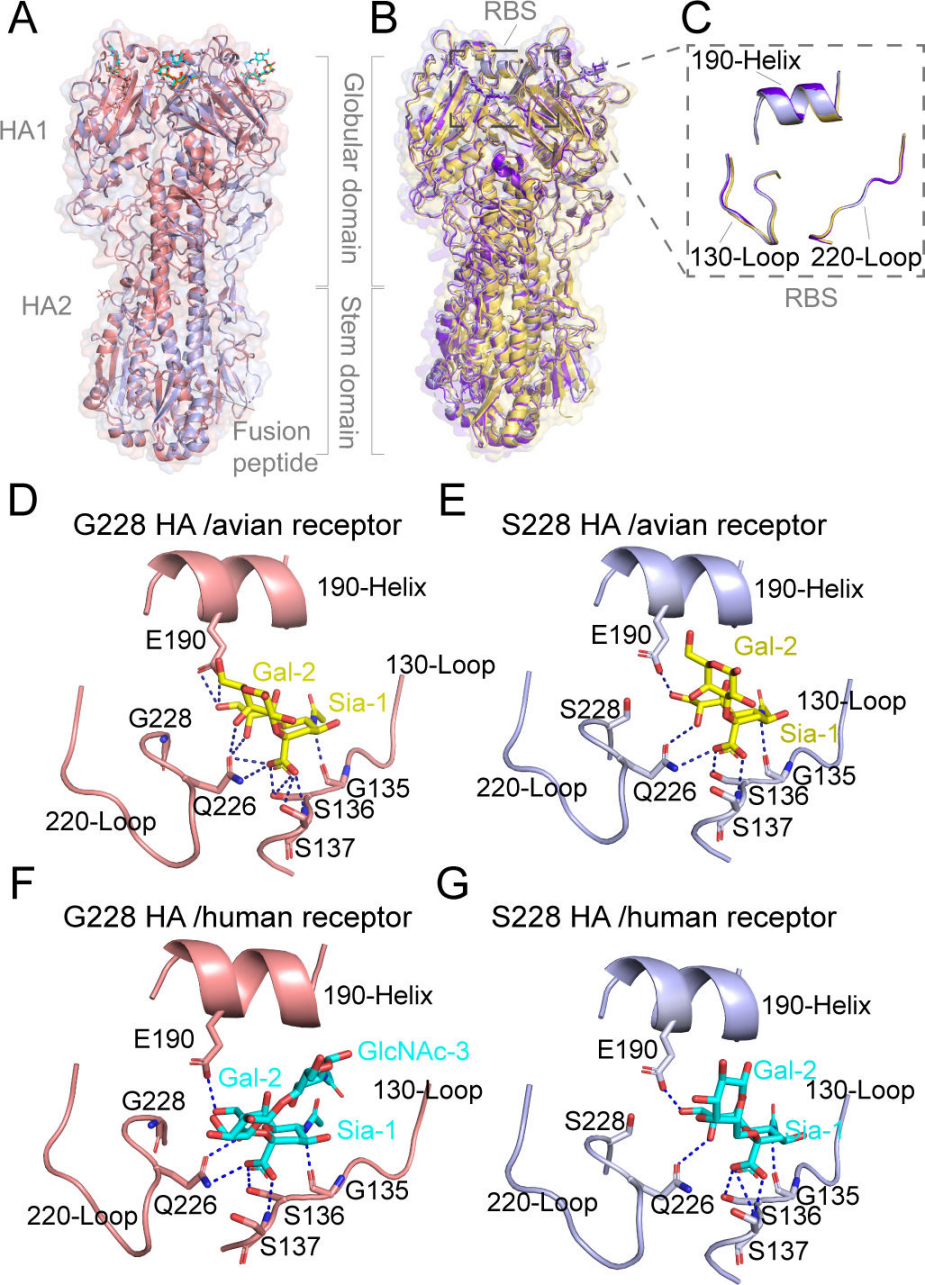
Overall structure and molecular interactions of human-origin HN/4-10 H3N8 HA with either avian or human receptor analogs. (**A**) Comparison of the overall structure of HN/4-10 H3N8 S228 and G228 HAs with human receptor analogs. H3N8 S228 HA is colored light blue, H3N8 G228 HA is colored salmon. (**B and C**) Comparison of the apo and complex structures with human or avian receptor analogs of the (**B**) total and (**C**) RBS of HN/4-10 H3N8-S228 HA. Apo H3N8-S228 is colored yellow, complex with human receptor analog is colored light blue and complex with avian receptor analog is colored purple blue. (**D and E**) H3N8 G228 and S228 RBS with avian receptor analogs. (**F and G**) H3N8 G228 and S228 RBS with human receptor analogs. H3N8-G228 (salmon) and H3N8-S228 (light blue) are shown in cartoon. Avian (yellow) and human (cyan) receptor analogs are shown in sticks. Hydrogen bonds between the HAs and the analogs are shown in dark blue.

Next, we solved the apo structure of the HN/4-10 H3N8 HA by X-ray (Table S1), and the complex structures with avian or human receptor analogs [LS-tetrasaccharide a (LSTa) and LS-tetrasaccharide c (LSTc)] by cryo-electron microscopy (cryo-EM) (Table S2; Fig. S2 to S6). The electron density for the ligands is unambiguous (Fig. S7). Comparative analysis of the overall structures between the G228 and S228 variants of HN/4-10 H3N8 HA revealed a remarkable similarity (Fig. 4A). Furthermore, the RBS in the apo state and in complex with receptor analogs displayed minimal structural alteration (Fig. 4B and C). In the structures of H3N8 G228 and S228 HAs complexed with the avian receptor analog, the receptor adopted a *trans* conformation and established robust and extensive interactions with RBS residues (Fig. 4D and E). These interactions included several conserved binding sites in H3N8 HA, such as E190 and G135 (Table S3). Importantly, the presence of the “avian signature” Q226 also played a vital role in binding to the sialic acid (SIA-1) and galactose (GAL-2) moieties within LSTa (Fig. 4D). Conversely, in the structures of H3N8 G228 and S228 HAs complexed with the human receptor analog LSTc, the receptor adopted a *cis* conformation, accompanied by a reduction in the number of contacts with the HAs compared to those of LSTa complex structures (Table S3). Nevertheless, both structures formed conserved hydrogen bonds with SIA-1 at residues G135/S136/S137/E190/Q226 (Fig. 4F and G). Notably, residue S228 in the S228 protein formed two weak hydrogen bonds (bond length 3.6 Å) with LSTc, whereas the interactions were notably absent in G228 (Fig. 4E and G; Table S3). These results suggested that the G228S substitution to some extent enhances the binding ability of the H3N8 HA protein to the human receptor, which is consistent with our SPR results.

### Structural comparison of HN/4-10 H3N8 and other H3 with avian or human receptors

**Fig 5:**
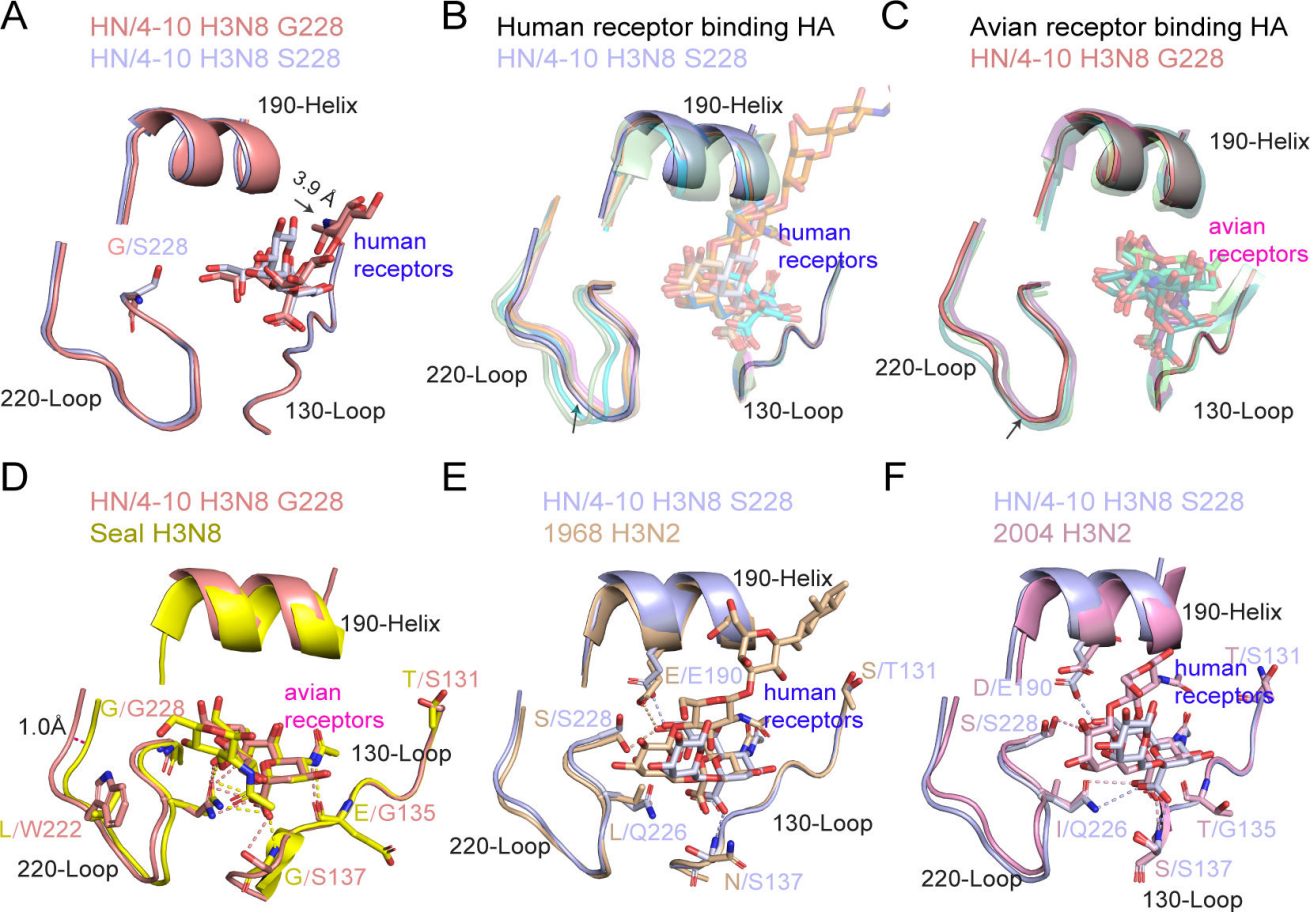
Comparison of RBS between HN/4-10 H3N8 and other H3 HAs. (**A**) Structural comparison of HN/4-10 H3N8 S228 and G228 RBS complex with human receptor analogs. (**B**) Comparison of HN/4-10 H3N8 S228 RBS with other H3 HAs that bind human receptor analogs. (PDB: 2YPG, 2YP3, 2YP8, 6AOV, 6BKT, 6BKR, 6NSB) (**C**) Comparison of HN/4-10 H3N8 G228 RBS with other H3 HAs that bind avian receptor analogs. (PDB: 4UNX, 4UO1, 4UO5, 4WA2). H3N8 S228 and G228 HAs were indicated by the arrows, respectively. (**D**) Comparison of HN/4-10 H3N8 G228 and Seal H3N8 complex with avian receptor analogs (Seal H3N8 PDB: 4WA2). (**E**) Comparison of HN/4-10 H3N8 S228 and 1968 H3N2 complex with human receptor analogs (1968 H3N2 PDB: 2YPG). (**F**) Comparison of HN/4-10 H3N8 S228 and 2004 H3N2 complex with human receptor analogs (2004 H3N2 PDB: 2YP3). HN/4-10 H3N8 S228 is colored light blue, G228 is colored salmon. Seal H3N8 is colored gray, 1968 H3N2 is colored wheat, and 2004 H3N2 HA is colored light pink.

We examined the RBS structures of S228 and G228 HA variants after binding to the human receptor analog LSTc. The RBS structures of S228 and G228 HAs were highly similar, with the S228 RBS being slightly larger than the G228 RBS (Fig. 5A). However, a significant difference was observed in the orientation of the sugar ring, where the C1-C2 atoms of the G228 GAL-2 were rotated outward by about 40° compared to the S228, resulting in its GAL-2 being about 3.9 Å further away from the 190-helix than the S228. We then compared the HN/4-10 H3N8 HA complex structures with all known H3 structures (Fig. 5B and C; Fig. S8). In general, for human-receptor-binding H3, the distance of the 220 Loop from the 130 Loop of HN/4-10 H3N8 S228 fell within the middle range (Fig. 5B). Notably, the largest distance was observed in the case of the 2011 H3N2 HA, followed by the A/Hong Kong/4443/2005 H3N2 (2005 H3N2) HA (19) (Fig. 5B; Fig. S8A and E). This may be attributed to the presence of the less hydrophobic Glu226 residue in HN/4-10 H3N8 compared to Ile and Leu in the aforementioned HAs. The SIA-1 and GAL-2 of HN/4-10 H3N8 are less vertical compared to other H3N2-bound sugar rings (Fig. 5B). In detail, compared with 1968 H3N2, the RBS of HN/4-10 H3N8 was smaller, which was a consequence of the Q226L substitution in 1968 H3N2, leading to enhanced hydrophobic interactions and higher binding affinity to LSTc (Fig. 5E). Moreover, HN/4-10 H3N8 is less like 2004 H3N2 than to 1968 H3N2 (Fig. 5F). 2004 H3N2 has three substitutions, E190D, G225D, and L226I, which result in reduced binding affinity for human receptor (19). Besides, the RBS of HN/4-10 H3N8 is wider than that of 2004 H3N2 (Fig. 5F), implying an evolutionary adaptation for improved binding to human receptors compared to the 2004 H3N2 strain. We speculate that the G137S substitution may enhance the interaction between the hydroxyl of serine and human receptor analogs.

For all HA structures of H3 that bind avian receptors, including the HA of H3N8 of canine, equine, and seal origin, the distance between the 220 Loop and 130 Loop is similar, except for the A/eq/Newmarket/93 /H3N8 (PDB: 4UNX) (23), which is slightly larger at 221-224 region (Fig. 5C; Fig. S8F). we found that the binding pattern of HN/4-10 H3N8 G228 RBS to avian receptors closely resembled that of Seal H3N8, an HA with exclusive avian receptor-binding capability (24) (Fig. 5D). Nevertheless, it is worth noting that the RBS of HN/4-10 H3N8 G228 was approximately 1 Å larger than that of Seal H3N8 (Fig. 5D). These variations in receptor binding can likely be attributed to the L222W substitution in HN/4-10 H3N8 (Fig. 1B and 5D). We speculate this substitution serves as a pivotal determinant for HN/4-10 H3N8 to acquire the ability to bind to human receptors, as it likely enhances the hydrophobic interactions between the HA protein and human receptors. These findings strongly imply that HN/4-10 H3N8 has developed the capability to bind to human receptors, marking a critical milestone in its adaptation to the human host.

### The Q226L mutant changed the receptor-binding preference of S228 and G228

**Fig 6:**
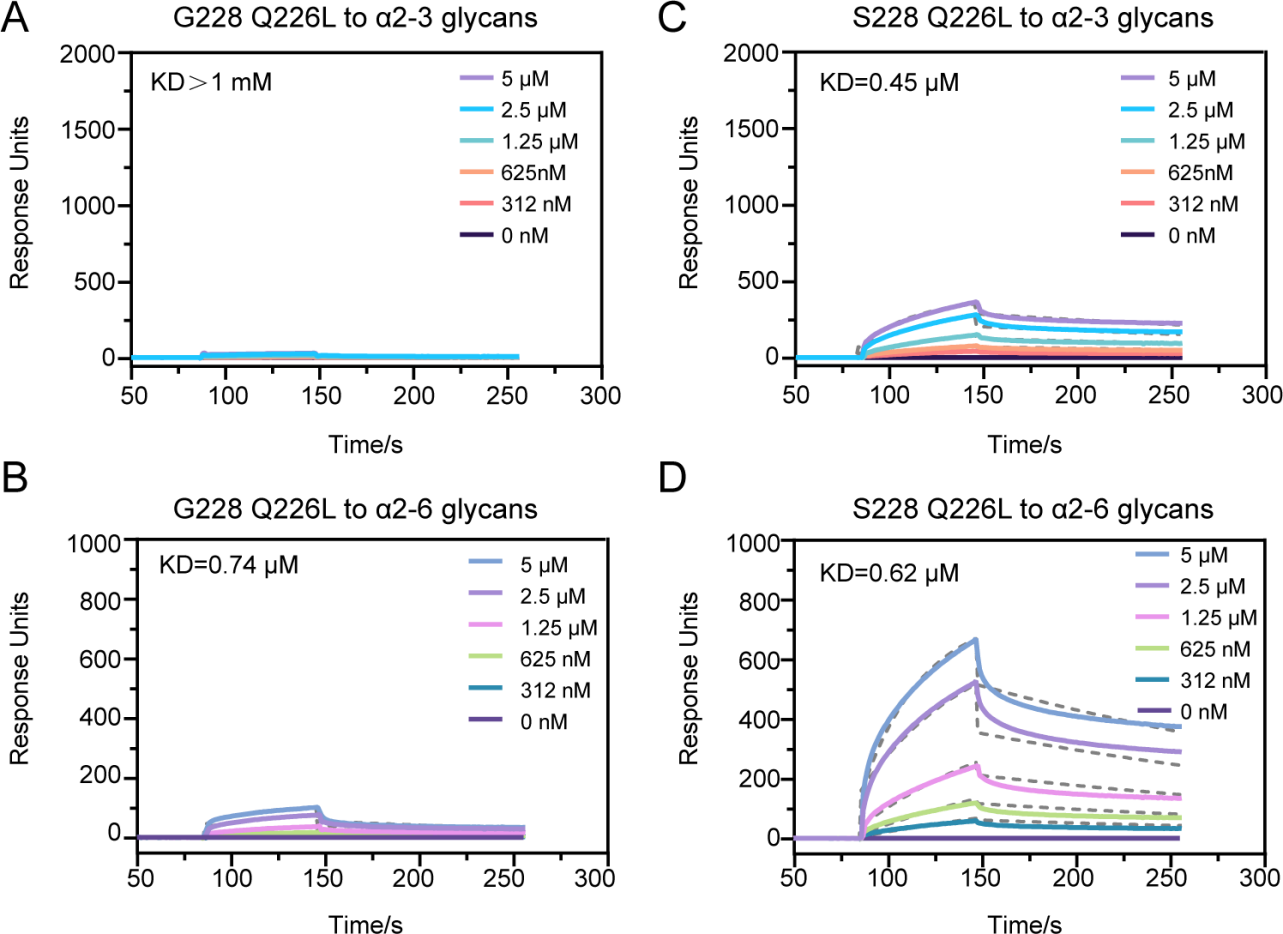
Receptor-binding properties of H3N8 G228 and S228 Q226L HA. (**A and B**) BIAcore diagrams of G228 Q226L mutant binding to the α2-3-linked and α2–6-linked sialylglycan receptors. (**C and D**) BIAcore diagrams of S228 Q226L mutant binding to the α2-3-linked and α2-6-linked sialylglycan receptors. The plots and K_D_ values are one representative data set from three independent experiments. Dashed lines represent the data fitted by BIAevaluation software 4.1 using a 1:1 Langmuir binding model.

Although the HN/4-10 H3N8 strain already possesses the adaptive mutation G228S, it retains the Q226 residue. For IAVs such as H3N2 and H4N6, L226 has been identified as favorable for binding to α2-6 linked sialic acid (SA), and the Q226L mutation is commonly observed in the HA of influenza viruses adapted for human infection (9, 38). To investigate whether the Q226L mutation alters the receptor-binding properties of H3N8 HA, we introduced this mutation into both the S228 and G228 HA. In the SPR analysis, the Q226L mutation significantly reduced the affinity of H3N8 HA for avian receptor. Specifically, for the G228 variant, no binding response was detected, while the S228 variant exhibited a fourfold reduction in affinity (Fig. 6A and C). Conversely, the Q226L mutation markedly increased the affinity for human receptor (Fig. 6B and D). The affinity of both G228 and S228 for human receptor increased more than 10-fold, with S228 showing stronger binding than G228. These findings are consistent with our previous observation that the G228S mutation has a significant effect on HA’s affinity for human receptor and indicates that the Q226L mutation can shift H3N8 HA’s receptor-binding preference from avian to human. Notably, the Q226L mutation on the G228 background results in significantly lower binding responses, indicating an incompatibility between the amino acids at these two positions.

### The major antigenic and glycosylation sites of HN/4-10 H3N8 HAs are distinct from other H3

**Fig 7:**
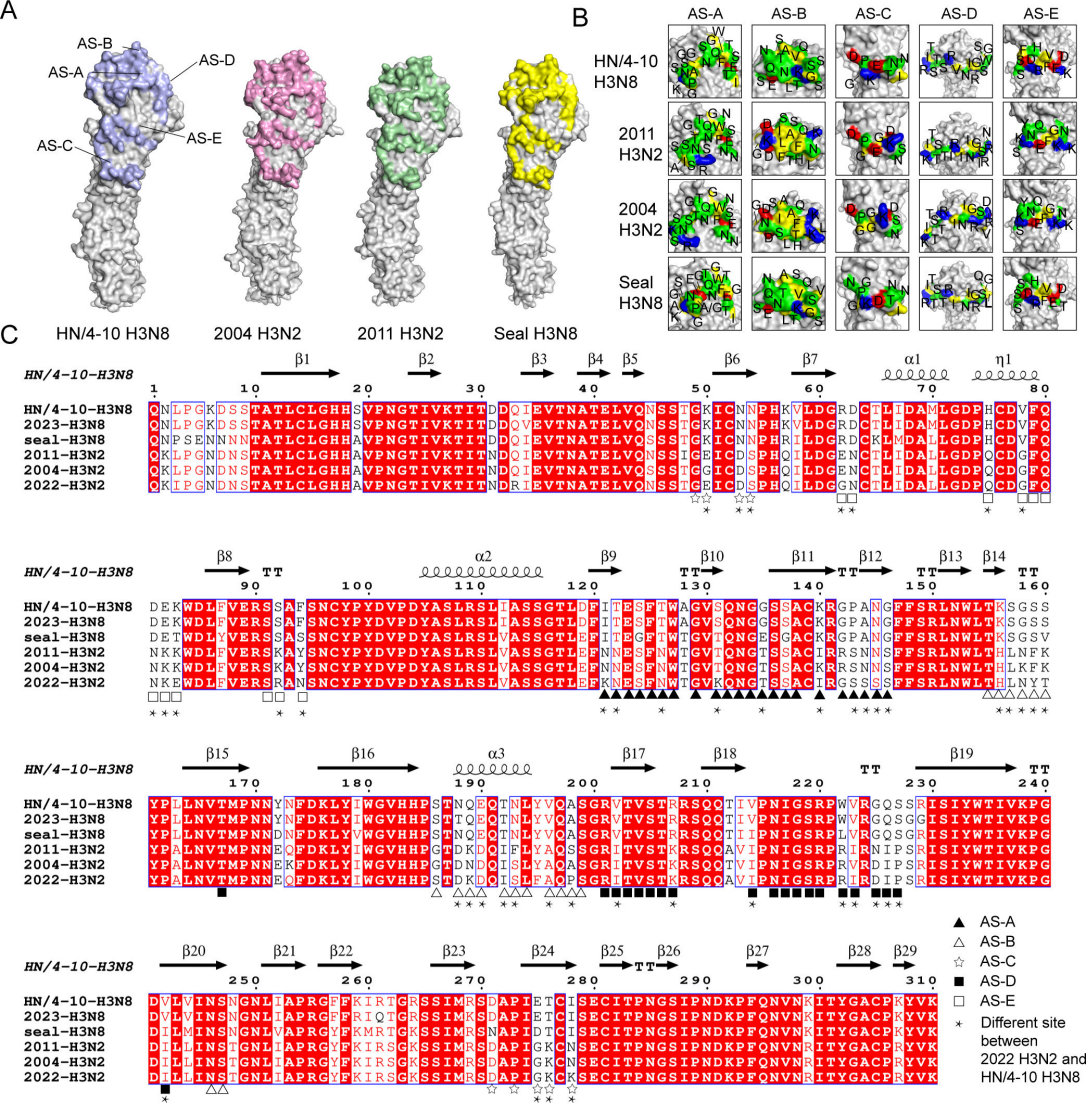
Comparison of antigenic sites of HN/4-10 H3N8 and other H3 HAs. (**A**) General locations of antigenic sites of HN/4-10 H3N8, human 2004 H3N2 (PDB: 2YP2), 2011 H3N2 (PDB: 4WE8), seal H3N8 (PDB: 4WA2) HAs. HA molecules are shown as surface representations. (**B**) Comparisons of antigenic sites of HN/4-10 H3N8 HA and another three H3 HAs. Amino acids are colored as follows: positive (Arg and Lys), blue; negative (Asp and Glu), red; hydrophobic (Ala, Phe, Gly, Ile, Leu, Met, Val, and Trp), yellow; polar (His, Asn, Gln, Ser, Thr, Pro, and Tyr), green. (**C**) Sequence alignment of HN/4-10 H3N8 HA with seal H3N8, 2004 H3N2, 2011 H3N2, 2022 H3N2, and 2023 H3N8 (41).

Five classic antigenic sites (AS) locate on the surface of H3 subtype HA (39, 40). We compared the HA surface AS of H3N8 S228 HA with those of Seal H3N8, 2004 H3N2, 2011 H3N2, 2022 H3N2. Although the AS distributions are generally similar (Fig. 7A), the AS are not conserved, and the main differences are at the AS-A and AS-B sites (Fig. 7B and C). Changes in surface antigenic sites enable the virus to evade the immune system and elude the protection of vaccines and antibodies. The amino acid of the AS of HN/4-10 H3N8 HA were significantly different from those of H3N2, suggesting that seasonal influenza vaccination may not effectively protect against H3N8 infection. Consistent with this analysis, the previous serological investigation for antibodies to IAVs among poultry workers in HN/4-10 and Hunan provinces also found no reaction for H3N8 but 32% seropositive for human H3N2 inﬂuenza (31). N-linked glycosylation (NXT/S) analysis and 3D structures revealed that the human-derived H3N8 HA had significantly fewer glycosylation sites compared to the 2004 H3N2 (PDB:2YP2) (19) and 2011 H3N2 (PDB:4WE8) (16) (Fig. S9). Only six glycosylation sites were found in HN/4-10 H3N8 HA, located at positions 22, 38, 45, 165, 285, and 483 (Fig. S9A). The modified glycosylation patterns may also contribute to the alteration in antigenicity of H3N8 HA.

## DISCUSSION

There have been five human influenza pandemics in the last 108 years, the most devastating being the 1918 pandemic caused by the H1N1 virus, which killed more than 40 million people (42). Influenza viruses continue to pose a significant threat to human health today (43). The H3N2 IAV has been introduced into humans since 1968 and has caused numerous seasonal epidemics. Many other H3Ny subtypes, in addition to H3N2, have long been circulating among mammals (25, 44, 45). This suggests the potential for future human infections with H3Ny subtypes (46). Previous studies have found that the seal H3N8 HA was able to bind to human and ferret lung tissues, but not tracheal tissues, and showed little binding to human receptor analogs (22, 24). While recent H3N8 viruses exhibited lower virulence compared to the 2009 pandemic H1N1 (09pdmH1N1) viruses, they elicited comparable levels of infectivity in mice (33). Most importantly, the Henan human-derived H3N8 we studied here is capable of droplet transmission in ferrets (35, 47). All the results illustrate the evolution of H3N8 IAVs and the significant risk of further adaptation to humans.

Amino acid mutations in HAs can alter the binding preference of HA for human and avian receptors. In previous studies, the D225G/ E190D mutations shifted H1 from early dual receptor binding to human receptor preference binding (48), and the Q226L/G228S mutations changed H2 and H3 from avian receptor specific binding to human receptor preference binding (9). Avian IAVs, such as H7 and H10, have not fully adapted to humans yet. However, amino acid substitutions in the RBS of their HAs still enable them to bind to human receptors, resulting in significant damage (49). For all other isolated H3N8 HAs, the key residue at position 228 remained G. However, in the HN/4-10 H3N8 strain, it could be translated as either G or S due to codon degeneracy, suggesting an ongoing evolution toward a human receptor binding preference. In our study, we found that the naturally occurring G228S mutation plays a crucial role in altering the receptor-binding properties of H3N8 HA for both human and avian receptors. The HA of the S228 H3N8 variant shows a stronger adaptation for binding to human receptors compared to the G228 variant. Nevertheless, the HN/4-10 H3N8 strain studied here remains predominantly an avian influenza virus and has not yet fully adapted to humans, as it retains Q at position 226 in its HA. To further investigate, we confirmed that the Q226L mutation shifts the HA’s binding preference from avian to human receptors, and the binding response values for Q226L on G228 are significantly lower than on the S228 background, indicating that the Q226L mutation on G228S may pose a great threat to human health. Similarly, another study found that virus transmission via respiratory droplets occurred in ferrets infected with the A/Henan/2022 virus carrying HA G228 (47). Deep sequencing of nasal swab samples from both inoculated and exposed ferrets revealed sequence polymorphisms in the HA protein, including HA-226L, HA-228S, or HA-228A mutations, indicating the importance of these mutations. Moreover, our previous findings indicate that the HA-G228S and PB2-E627K substitutions are key determinants for the airborne transmission of the HN/4-10 virus in ferrets (35). These findings suggest ongoing H3N8 influenza virus adaptation to mammalian hosts, highlighting the dynamic nature of influenza viruses in their quest for host fitness.

The surface antigen profile of HA is directly related to human immunity. The effectiveness of influenza vaccines has declined in recent years, particularly against strains of the H3N2 subtype (2). Previous studies have also shown that sera from people vaccinated against seasonal influenza are naïve to chicken and human H3N8 IAVs (33, 50). We compared the amino acid of surface AS of HN/4-10 H3N8 with 2004 H3N2, 2011 H3N2, Seal H3N8, and 2022 H3N2 HAs. We found that it shares common antigenic epitopes with seal H3N8, but with low similarity to H3N2. Therefore, we suspect that H3N8 viruses capable of infecting humans may not be well protected by the current H3N2-based vaccine or H3N2 post-infection. However, research has also found that human sera possess cross-reactive antibodies to the reassortant H3N8 virus, particularly in older donors (47), possibly due to geographic location or differences in strains and timing of infection. H3Ny subtype IAV also evades immune pressure through N-linked glycosylation sites on the globular head. Compared to H3N2, HN/4-10 H3N8 has significantly fewer N-glycosylation sites than recent circulating H3N2. Despite being “a single case,” the reported first death case indicates that the threat posed by the virus cannot be underestimated.

In conclusion, we investigated the receptor-binding properties of the first human-infecting H3N8 HA and found that it has a dual receptor-binding capacity with preference for avian receptor binding, and the G228S substitution slightly increased the binding to human receptors. Structural analysis showed that its RBS was enlarged, and the antigenic epitope of HN/4-10 H3N8 also shows a major change compared to the circulating H3N2 strains, indicating a risk that HN/4-10 H3N8 will continue to evolve and infect more humans. This deserves further surveillance work in the future.

## MATERIALS AND METHODS

### Phylogenetic tree construction and sequence alignment

The phylogenetic tree and multiple sequence alignments (MSAs) of full length HAs were generated using MEGA (version 11.0.13) by Maximum likelihood and ClustalW methods. Sequence alignment of HN/4-10 H3N8 HA (accession number EPI2024823) with A/Guangdong/ZS-23SF005/2023 (2023 H3N8) (accession number EPI2508604), A/harbor seal/Massachusetts/1/2011 (seal H3N8) (accession number EPI382073), A/Finland/486/2004 (2004 H3N2) (accession number EPI397685), A/Victoria/361/2011 (2011 H3N2) (accession number EPI2120562), and A/Hunan-Yuhua/11022/2022 (2022 H3N2) (accession number EPI2125428) was generated by ENDscript server (version 2.0) (41).

### Gene cloning, expression, and protein purification

The sequences encoding A/Henan/4-10/2022 H3N8, A/Kansas/14/2017 H3N2 (2017 H3N2) (accession number EPI1691978), and A/Indonesia/5/2005 H5N1 (InH5) (accession number EPI116487) HAs were synthesized and cloned into the pFastBac1 vectors. Specifically, because of the degeneracy of the protein at residue 228, two clones were designed as S228 and G228. An N-terminal GP67 signal peptide for secretion and thrombin cleavage site, trimer tags, and His6 tag were added to the C-terminus for purification. The Q226L mutant protein constructs were designed in the same manner as the other HAs.

HAs were produced by infecting Hi5 cells for 48 hours. Supernatants were collected and soluble proteins were recovered by metal affinity chromatography using HisTrap HP 5 mL columns (Cytiva). HAs were then dialyzed into lower salt buffer (20 mM Tris, 20 mM NaCl, pH 8.0) and further purified by ion exchange chromatography. Finally, gel filtration chromatography was performed on a HiLoad 16/600 Superdex 200 pg column (Cytiva) in PBST (10 mM Na_2_HPO_4_, 1.76 mM KH_2_PO_4_, 137 mM NaCl, 2.7 mM KCl, pH 7.4, 0.005% Tween20) buffer for SPR and 20 mM Tris, 150 mM NaCl, pH 8.0 buffer for crystallization and cryo-EM sample preparation.

### Crystallization, data collection, and structure determination

H3N8 S228 HA was crystallized by the sitting-drop vapor diffusion method at 18°C. The crystals were grown in a reservoir solution of 28% (vol/vol) 2-propanol, 0.1 M BIS-Tris pH 6.5, 3% (vol/vol) polyethylene glycol 200 for 5 days. After a short soak in the reservoir solution with the addition of 20% (vol/vol) glycerol, the crystals were rapidly cooled in liquid nitrogen. X-ray diffraction data were collected at the Shanghai Synchrotron Radiation Facility (SSRF) beamline 19U1 with 1.03961 Å radiation wavelength. The last resolution shell is 3.0 Å after processed and scaled with XDS (51).

The HA structure was solved by molecular replacement using 6WXB as a model with Phaser from the CCP4 program suite (version 9.0.005) (52). Refinement was performed using Phenix (version 1.20.1) (53). Extensive manual modeling adjustments were carried out in Coot (version 0.9.8) (54, 55). Subsequent refinement rounds were performed using the phenix.refine tool within the Phenix suite, which included rigid-body refinement, B-factor refinement, energy minimization, isotropic ADP refinement, and bulk solvent modeling, and the last resolution shell showing an Rfree value of 0.2779 at the last round of refinement. Final statistics for data collection and structure refinement, including resolution, R-factors, and validation metrics, are presented in Table S1. Hydrogen bonds and hydrophobic contacts were determined using the CONTACT of the CCP4 software suite.

### Cryo-EM sample preparation and data acquisition

For receptor analog complexes, 0.2 mg/mL HAs containing 5 mM LSTa (Accurate Chemical &Scientific Corporation, Cat# 55/50) or LSTc (Accurate Chemical & Scientific Corporation, Cat# 55/52) (56) were incubated for 4 h. Three microliters of purified H3 G228 or S228 complexed with LSTc or LSTa was applied to graphene oxide (GO) grids (GO on Quantifoils R1.2/1.3 300 mesh copper grids, R1.2/1.3). The blotted grids (2 s, 100% humidity and 4°C) were rapidly frozen in liquid ethane (Vitrobot Mark IV, Thermo Fisher Scientific). The movie stacks were collected on a 300 kV Titan Krios transmission electron microscope equipped with a Gatan K3 detector and GIF Quantum energy filter in super-resolution counting mode (magnification of 105,000×, physical pixel size of 0.85 Å) using EPU. Each movie was dose-fractionated into 32 frames with a total dose of 50 e-/Å2 (defocus range of -1.0 to -2.0 µm).

### Image processing, 3D reconstruction, model building, and structure refinement

The data sets were processed using cryoSPARC (version 4.1.0) (57). Details are shown in the supplemental material (Fig. S2 to S5).

For S228 complexed with LSTc, 5,727 raw movies were motion corrected using MotionCor2 (version 1.2.4) (58). The contrast transfer function (CTF) parameters were estimated using CTFFIND4 (59). Blob picking in a subset of 1,000 yielded 620,082 particles, and 2D classification separated out 213,620 clean particles for template picking. Template picking yielded a total of 1,493,140 particles which were extracted and subjected to 2D classification. After 2D classification, 1,063,056 clean particles were selected to perform initial reconstruction and heterogeneous refinement, which separated out 666,079 particles. After homogeneous refinement with C1 symmetry, the final map was reconstructed at 2.09  Å based on the FSC using the 0.143 criterion. The image-processing workflow is summarized in Fig. S2.

For G228 complexed with LSTc, 4,724 raw movies were motion corrected using MotionCor2 (version 1.2.4) (58). The CTF parameters were estimated using CTFFIND4 (59 ). Blob picking in a subset of 500 yielded 318,102 particles, and 2D classification separated out 124,289 clean particles for template picking. Template picking yielded a total of 7,037,513 particles which were extracted and subjected to 2D classification. After 2D classification, 2,031,208 clean particles were selected to perform two rounds of initial reconstruction and heterogeneous refinement, which separated out 533,492 particles and yielded a 2.26 Å volume after non-uniform refinement (60). To improve the quality of ligand density, we applied masked 3D classification and a subset containing 22,596 particles were selected and subjected to non-uniform refinement (60) with C1 symmetry. The final map was reconstructed at 2.84 Å based on the FSC using the 0.143 criterion. The image-processing workflow is summarized in Fig. S3.

For S228 complexed with LSTa, 6,397 raw movies were motion corrected using MotionCor2 (version 1.2.4) (58). The CTF parameters were estimated using CTFFIND4 (59) . Blob picking in a subset of 500 yielded 307,180 particles, and 2D classification separated out 54,791 clean particles for template picking. Template picking yielded a total of 10,983,162 particles which were extracted and subjected to 2D classification. After 2D classification, 591,818 clean particles were selected to perform initial reconstruction and heterogeneous refinement, which separated out 250,384 particles. Homogeneous refinement with C1 symmetry and global CTF refinement pushed the resolution to 2.75 Å determined by the FSC = 0.143 criterion. The image-processing workflow is summarized in Fig. S4.

For G228 complexed with LSTa, 4,423 raw movies were motion corrected using MotionCor2 (version 1.2.4) (58). The CTF parameters were estimated using CTFFIND4 (59). Blob picking in a subset of 500 yielded 299,527 particles, and 2D classification separated out 53,021 clean particles for template picking. Template picking yielded a total of 6,666,324 particles which were extracted and subjected to 2D classification. After 2D classification, 570,647 clean particles were selected to perform initial reconstruction and heterogeneous refinement, which separated out 279,549 particles. Homogeneous refinement with C3 symmetry and global CTF refinement pushed the resolution to 2.09 Å determined by the FSC = 0.143 criterion. The image-processing workflow is summarized in Fig. S5.

Initial H3 models (PDB: 6WXB/7ZJ6) were fitted into the cryo-EM maps using UCSF Chimera (version 1.15) (61). The model was manually mutated and refined in Coot (version 0.9.8) (54, 55). Automated refinement was performed using Phenix (version 1.20.1) (53) with secondary structure and geometry restraints. Molprobity (62) was used to validate the geometry and evaluate the structural quality.

### SPR experiments

The affinities and kinetics of H3N8 HA binding to avian and human receptor analogs were analyzed using the BIAcore3000 system with streptavidin chips (SA chips, GE Healthcare). The biotinylated α2-6 glycans (6'SLNLN-b: NeuAcα2-6Galβ1-4GlcNAcβ1-3Galβ1-4GlcNAcβ1-SpNH-LC-LC-biotin) and the α2-3 glycans (3'SLNLN-b: NeuAcα2-3Galβ1-4GlcNAcβ1-3Galβ1-4GlcNAcβ1-SpNH-LC-LC-Biotin) were kindly provided by the Consortium for Functional Glycomics (Department of Molecular Biology, The Scripps Research Institute) and immobilized on the chip. Approximately 300 response units of biotinylated glycans were immobilized, and a blank channel was set as a negative control. HAs were serially diluted to different concentrations using PBST and flowed through the chip at a flow rate of 30 µL/min. The results were analyzed using BIAevaluation (version 4.1). Binding data of H3N8 HA and H3N2 HA were fitted using a 1:1 Langmuir binding mode, and affinity values were calculated using a simultaneous kinetic *K*a (association rate) / *K*d (dissociation rate) model. The affinity value of InH5 HA was calucated using a steady state affinity model due to the fast *K*a and *K*d. All experiments were repeated three times and representative plots are shown.

### Immunofluorescence staining assays

The immunofluorescence assays were performed as previously described (38). Briefly, paraffin-embedded human tracheal and duck small intestinal tissue sections were deparaffinized, rehydrated, and incubated overnight with 10% goat serum in PBS. Purified HAs with trimer tag and His6 tag at the C-terminus mixed with mouse anti-His tag (MBL, Cat# D291-3) and goat anti-mouse IgG Alexa Fluor 488 (Invitrogen, Cat# A28175) antibodies in a molar ratio of 4:2:1 were incubated for 20 min on ice and applied to tissues for 3 h incubation at room temperature. After three washes with PBS, 4′,6-diamidino-2-phenylindole (DAPI) was added to the sections and incubated to detect nuclei. After washing, tissue sections were mounted and imaged using a TCS SP8 (Leica) laser scanning confocal microscope.

### Circular dichroism assay

S228 and G228 proteins were diluted to 0.2 mg/mL in PBS. CD spectra were measured from 20°C to 95°C using ChiraScan CD spectrometer (Applied PhotoPhysics), increasing the temperature at 1°C/min. Thermal denaturation curves were determined by monitoring the CD value at 218 nm. Data were analyzed using GraphPad Prism 9 software.

## Data Availability

The atomic coordinates and cryo-EM density map and atomic coordinates have been deposited to the Protein Data Bank (PDB) and the Electron Microscopy Data Bank (EMDB): HN/4-10 H3N8 HA S228 complex with the avian receptor (PDB ID 8ZW6 and EMD-60517), S228 complex with the human receptor (PDB ID 8ZW7 and EMD-60518), G228 complex with the avian receptor (PDB ID 8ZW5 and EMD-60516), G228 complex with the human receptor (PDB ID 8X8R and EMD-38157). The crystal structure of S228 has been deposited to the PDB (PDB ID 8ZYK).

## SUPPLEMENTAL MATERIAL

Supplemental material jvi.01065-24-s0001.pdf Figures S1 to S9; Tables S1 to S3.

ASM does not own the copyrights to Supplemental Material that may be linked to, or accessed through, an article. The authors have granted ASM a non-exclusive, world-wide license to publish the Supplemental Material files. Please contact the corresponding author directly for reuse.
