# Supplementary material for: Structural basis of receptor-binding adaptation of human-infecting H3N8 influenza A virus
Source: J Virol. 2025 Feb 24;99(3):e01065-24. doi: 10.1128/jvi.01065-24 (PMC11915789; doi:10.1128/jvi.01065-24)
Supplement: Supplemental material — Figures S1 to S9; Tables S1 to S3. [file jvi.01065-24-s0001.pdf]

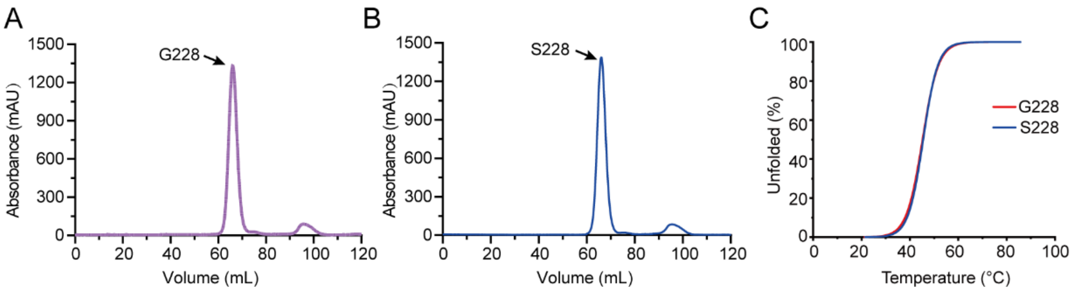

Figure S1. Purification and thermostability analysis of HN/4-10 H3N8 S228 and G228 HAs.

(A and B) Purification of G228 and S228 HAs by HiLoad™ 16/600 Superdex™ 200 pg. (C) The thermostability of HN/4-10 H3N8 S228 and G228 HAs. The midpoint transition temperatures (Tm) of S228 and G228 were 45.4 °C and 45.2 °C, respectively.

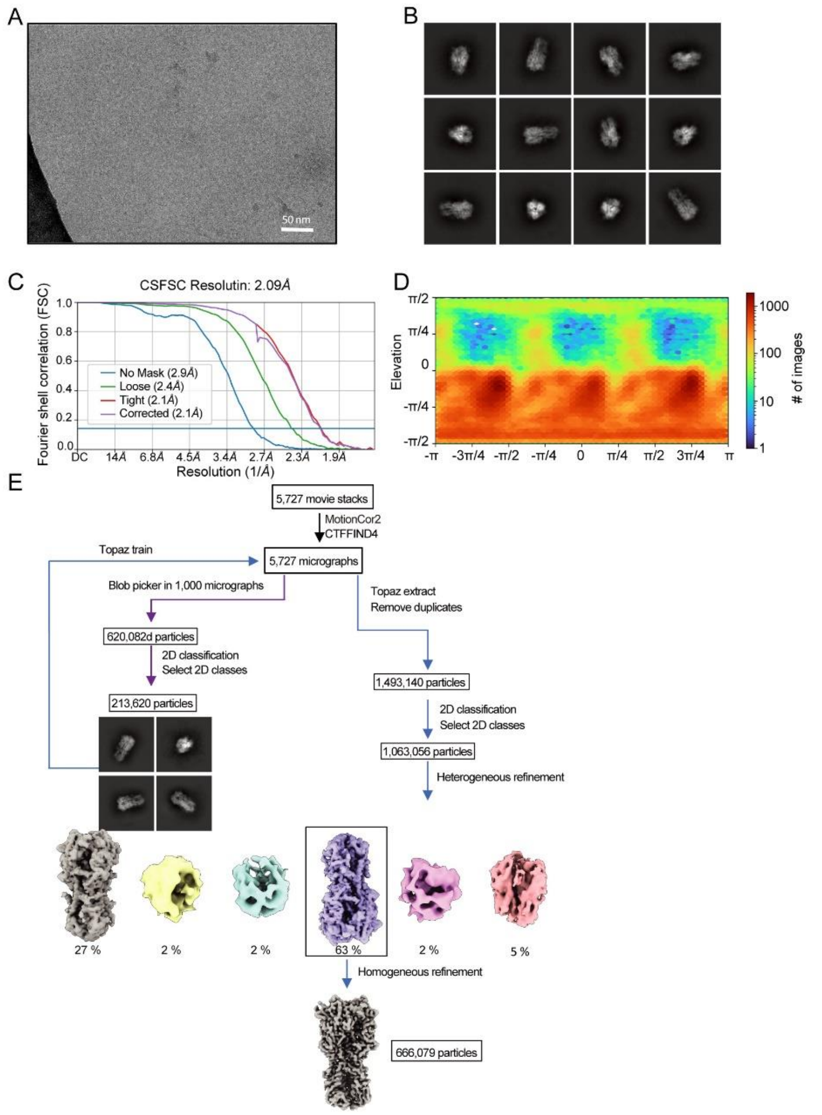

Figure S2. Cryo-EM single-particle analysis of HN/4-10 H3N8 S228 HA/LSTc complexes

(A) Representative cryo-EM micrographs collected for the H3N8 S228 HA/LSTc complex. (B) 2D class mean images. (C) The gold-standard Fourier shell correlation (FSC) curve for the density map at 2.09 Å resolution. (D) The viewing direction distribution plot for the H3N8 S228 HA/LSTc complex. (E) A brief workflow of cryo-EM image processing and reconstruction.

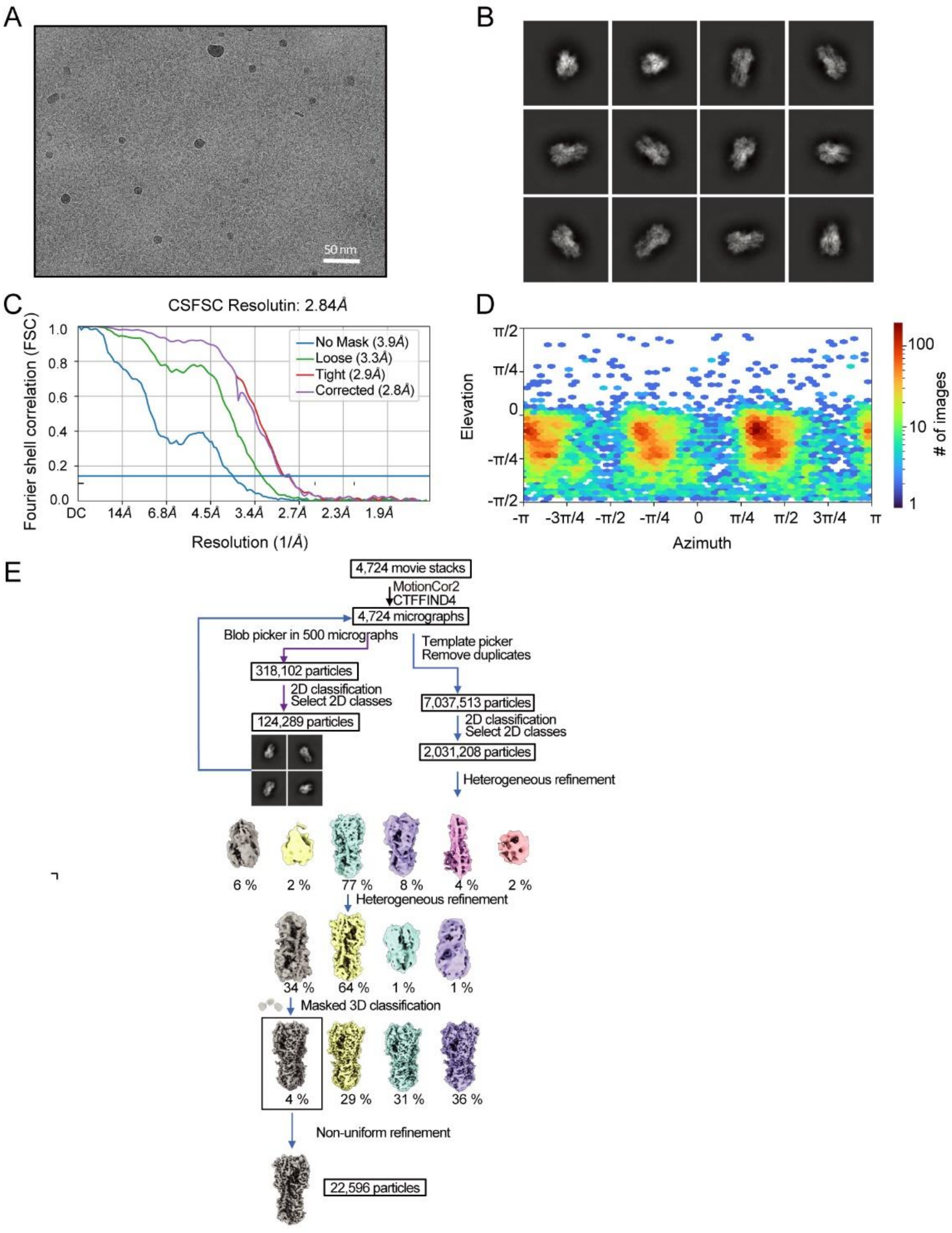

Figure S3. Cryo-EM single-particle analysis of HN/4-10 H3N8 G228 HA/LSTc complexes

(A) Representative cryo-EM micrographs collected for the H3N8 G228 HA/LSTc complex. (B) 2D class mean images. (C) The FSC curve for the density map at 2.84 Å resolution. (D) The viewing direction distribution plot for the H3N8 G228 HA/LSTc complex. (E) A brief workflow of cryo-EM image processing and reconstruction.

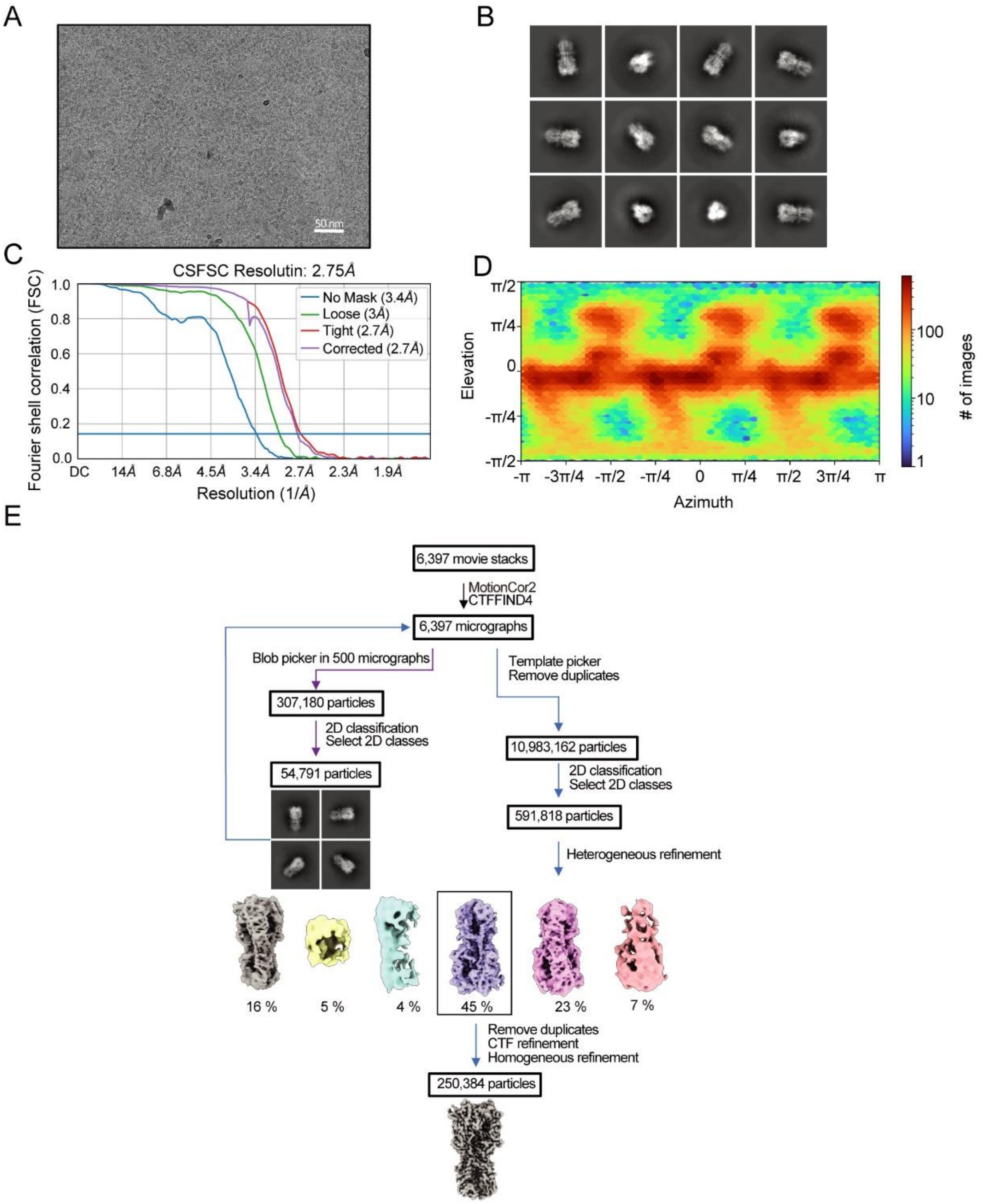

Figure S4. Cryo-EM single-particle analysis of HN/4-10 H3N8 S228 HA/LSTa complexes

(A) Representative cryo-EM micrographs collected for the H3N8 S228 HA/LSTa complex. (B) 2D class mean images. (C) The FSC curve for the density map at 2.75 Å resolution. (D) The viewing direction distribution plot for the H3N8 S228 HA/LSTa complex. (E) A brief workflow of cryo-EM image processing and reconstruction.

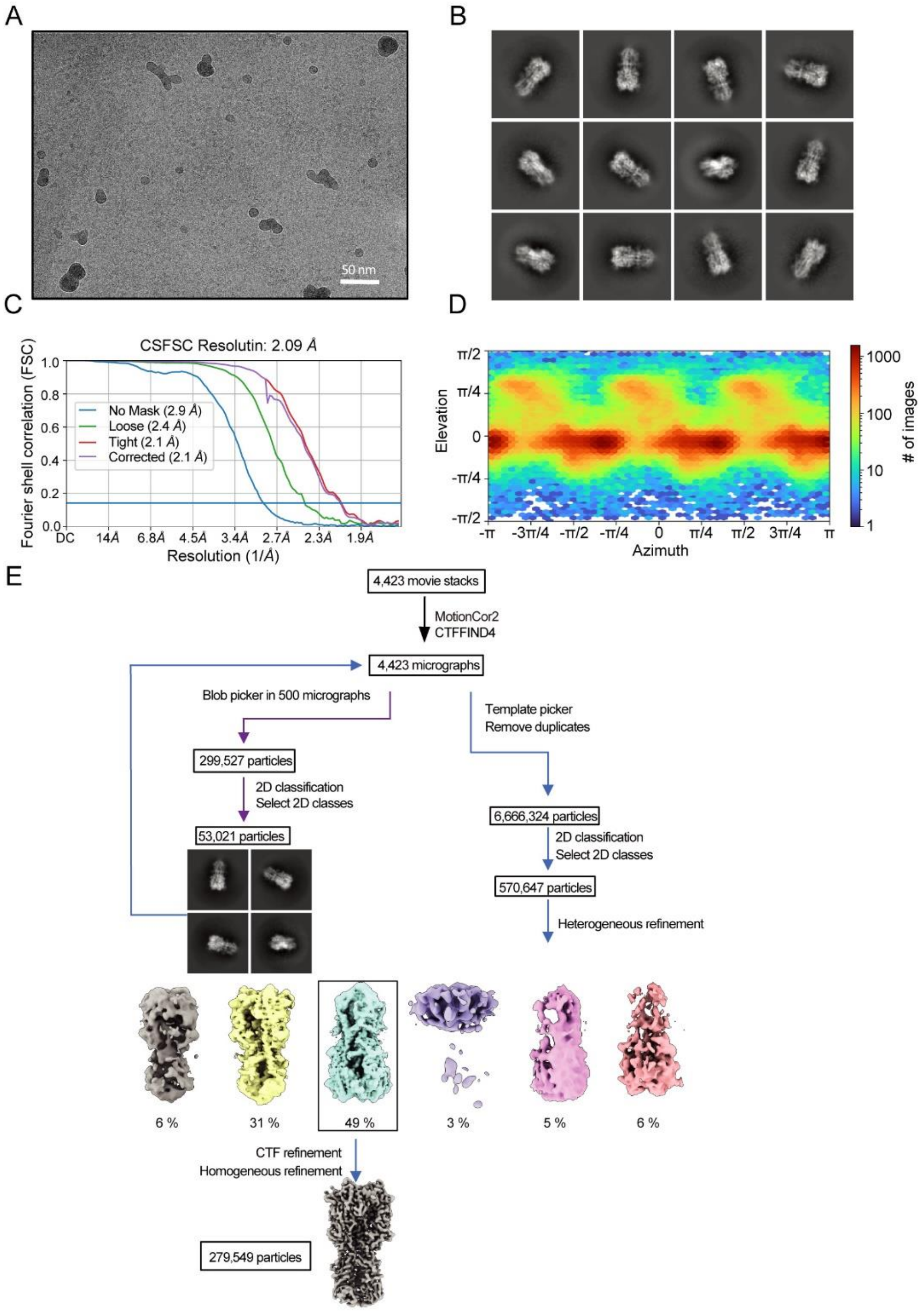

Figure S5. Cryo-EM single-particle analysis of HN/4-10 H3N8 G228 HA/LSTa complexes

(A) Representative cryo-EM micrographs collected for the H3N8 G228 HA/LSTa complex. (B) 2D class mean images. (C) The FSC curve for the density map at 2.75 Å resolution. (D) The viewing direction distribution plot for the H3N8 G228 HA/LSTa complex. (E) A brief workflow of cryo-EM image processing and reconstruction.

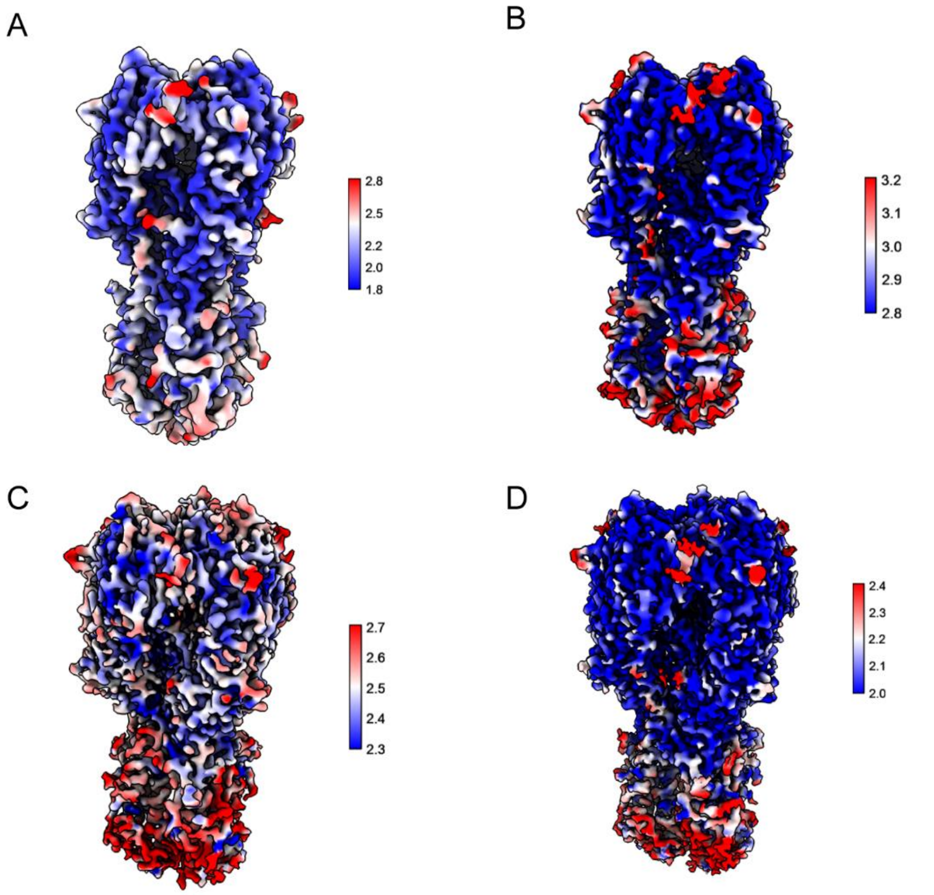

Figure S6. Cryo-EM maps local resolution of HN/4-10 H3N8 HA/receptor complex structures.

(A and C) Local resolution of S228 HA with avian and human receptors. (B and D) Local resolution of G228 HA with avian and human receptors. Surface is coloured according to the local resolution, low resolution colored in red and high resolution colored in blue, the resolution range for each complex is labeled. Figures were drawn using ChimeraX software.

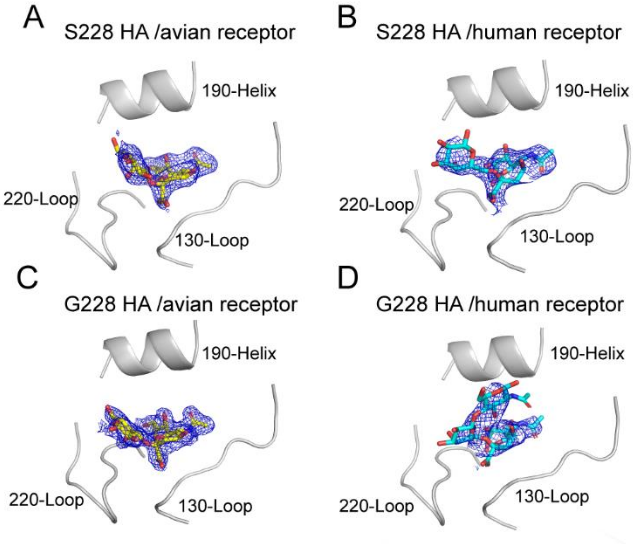

Figure S7. Cryo-EM density maps for the glycan receptors in the HN/4-10 H3N8 HA/receptor complex structures.

(A and B) Cryo-EM density maps for S228 HA with LSTc and LSTa. (C and D) Cryo-EM density maps for G228 HA with LSTc and LSTa. The panels show sections of the density maps for these glycan receptor analogs contoured at 1.0 sigma and 0.8 sigma, respectively, and the figures were drawn using PyMOL software.

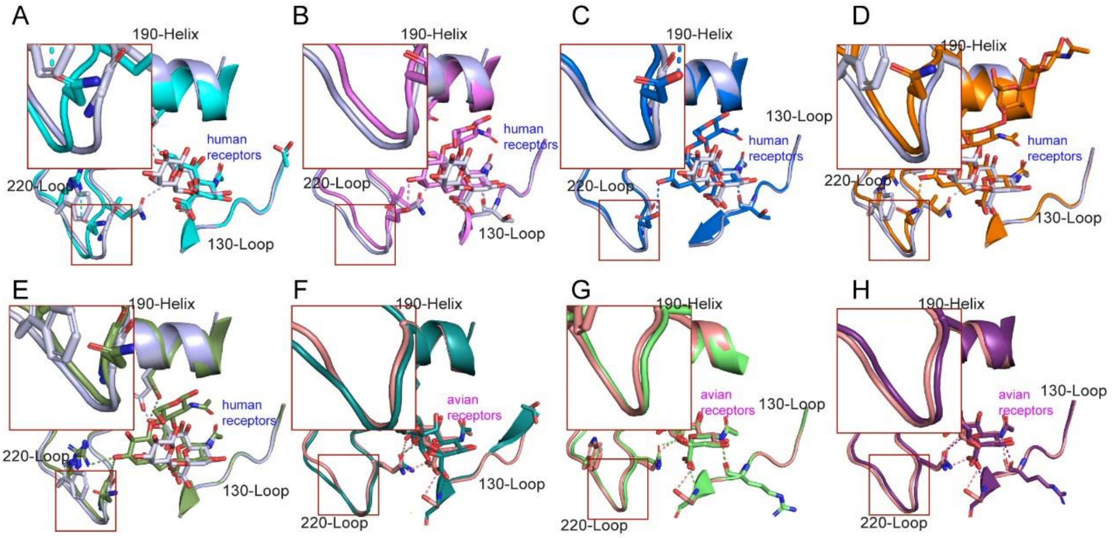

Figure S8. Comparisons of RBS between HN/4-10 H3N8 and other H3.

(A) Comparision of RBS region of HN/4-10 H3N8 S228 with 2005 Human H3N2 (PDB: 2YP8), colored cyan, (B) A/Michigan/15/2014 (H3N2), colored violet, (PDB: 6BKT), (C) A/Wyoming/3/2003 (H3N2) (PDB: 6BKR), colored marine, (D) A/Brisbane/10/2007 (H3N2) (PDB: 6AOV), colored orange, (E) A/Victoria/361/2011(H3N2) (PDB: 6NSB), colored smudge, (F) Comparision of RBS of HN/4-10 H3N8 G228 with A/eq/Newmarket/93/(H3N8) (PDB: 4UNX), colored deepteal, (G) A/canine/Colorado/17864/2006(H3N8) (PDB: 4UO5), colored lime, (H) A/equine/Richmond/1/2007(H3N8) (PDB: 4UO1) colored deep purple. All RBS structures were aligned of 130 loop and the magnified views shows the 220 loop differences in the H3N8 HA compared to other H3 HAs.

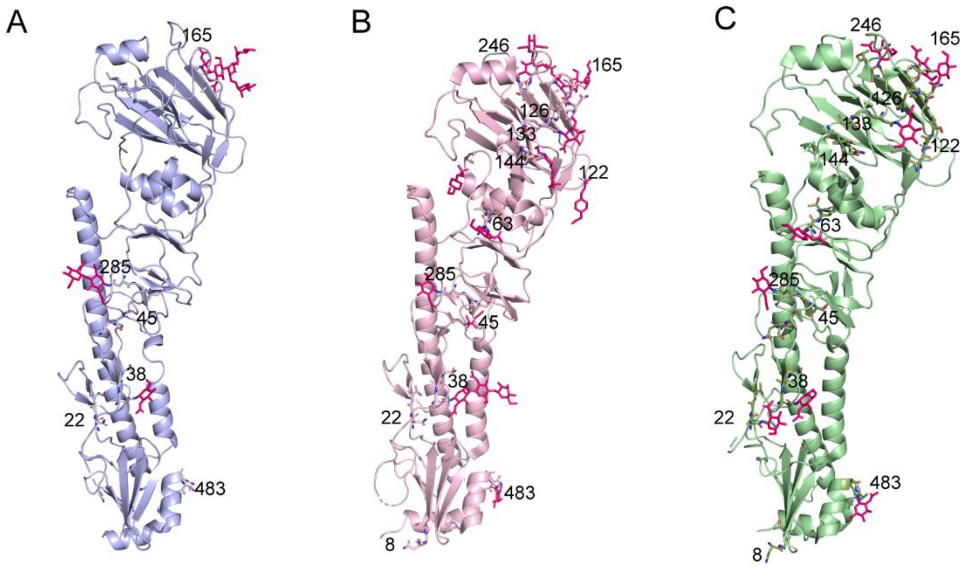

Figure S9. Potential glycosylation sites of different H3 HA

(A-C) HN/4-10 H3N8, 2004 H3N2 (PDB:2YP2) and 2011 H3N2 (PDB:4WE8) HA potential glycosylation sites are shown as sticks and labelled on the monomer. The visualized glycans in the structure are colored in deep pink.

Table S1. Crystallographic data collection and refinement statistics for HN/4-10 H3N8 S228 HAs.

	S228 HA
**Data collection**	
Space group	$P2_12_12_1$
Cell dimension	
a, b, c (Å)	136.829, 167.321, 206.841
α, β, γ (°)	90.0, 90.0, 90.0
Resolution (Å)	50.0 - 3.0
R_{merge}	0.271 (1.251)
$CC_{1/2}$	0.748 (0.703)
I / σI	8.3 (1.9)
Completeness (%)	99.9 (99.6)
Redundancy	10.0 (9.0)
**Refinement**	
No. reflections	93878
R_{work} / R_{free}	0.2380 / 0.2779
No. atoms	
Protein	23112
Ligand/ion	516
B-factors	
Protein	56.45
Ligand/ion	76.66
R.m.s. deviations	
Bond lengths (Å)	0.002
Bond angles (°)	0.48
Ramachandran plot	
Favored (%)	96.84
Allowed (%)	3.16
Outliers (%)	0.0
PDB accession code	8ZYK

Table S2. Cryo-EM data collection and validation statistics

	S228 / LSTc	G228 / LSTc	S228 / LSTa	G228 / LSTa
**Data collection and processing**				
Magnification	105K	105K	105K	105K
Voltage (kV)	300	300	300	300
Electron exposure (e–/Å^2)	50	50	50	50
Exposure rate (e-/pixel/s)	30	30	30	30
Defocus range (μm)	-1.0 ~ -2.0	-1.0 ~ -2.0	-1.0 ~ -2.0	-1.0 ~ -2.0
Pixel size (Å)	0.85	0.85	0.85	0.85
Symmetry imposed	C1	C1	C1	C3
Micrographs Collected (no.)	5,727	4,724	6,397	4,423
Final particle images (no.)	666,079	22,596	250,384	279,549
Map resolution (Å)	2.09	2.84	2.75	2.09
FSC threshold	0.143	0.143	0.143	0.143
**Refinement**				
Initial model used (PDB code)	6WXB/7ZJ6	6WXB/7ZJ6	6WXB/7ZJ6	6WXB/7ZJ6
Map sharpening *B* factor (Å^2)	74.4	76.2	117.2	70.5
Non-hydrogen atoms	11697	11629	11635	11629
Protein residues	1456	1448	1448	1448
Validation				
Clash score	2.32	3.66	2.07	1.90
Poor rotamers (%)	0.39	0.87	0.00	0.71
R.m.s. deviations				
Bond lengths (Å)	0.002	0.003	0.002	0.003
Bond angles (°)	0.404	0.442	0.430	0.524

Ramachandran plot				
Favored (%)	98.48	97.84	98.05	98.33
Allowed (%)	1.52	2.16	1.95	1.67
Outliers (%)	0.00	0.00	0.00	0.00
PDB accession code	8ZW7	8X8R	8ZW6	8ZW5

Table S3. Interaction between avian or human receptor analog and H3N8 HA

Receptor analog		H3N8 S228 HA	H3N8 G228 HA
LSTa	SIA	Y98(6), G134(2), G135(10,**1**), S136(15,**1**), S137(8,**1**), N145(1), W153(17), T155(1), E190(6,**1**), L194(6), Q226(5,**2**), S228(2), H183(3),	Y98(5,**2**), G134(2), G135(8,**1**), S136(10,**2**), S137(5,**2**), N145(1), W153(18), T155(2), H183(3,**1**), E190(9,**2**), L194(8), Q226(12,**3**), G228(1),
	GAL		E190(4), Q226(5,**1**)
Total		82,**6**	93,**14**
LSTc	SIA	Y98(3,**1**), G134(2), G135(10,**1**), S136(12,**1**), S137(7,**2**), N145(1,**1**), W153(18), T155(2), H183(4), E190(6,**1**), L194(4), Q226(6,**1**), S228(2)	Y98(8,**1**), G134(2), G135(9,**1**), S136(12,**1**), S137(9,**1**), N145(1), W153(18), T155(3), H183(4), E190(5,**1**), L194(9), Q226(8,**2**),
	GAL		
	NAG		N193(1), L194(1)
Total		78,**7**	90,**7**

Residues without underscores in parentheses indicate the number of vdw contacts between the receptor analogs and the H3N8 S228 or G228 HA. Underlined numbers in bold format indicate the number of H-bonds between the residues and the sugar ring. vdw contact was analyzed at a cutoff of 4.5 Å and H-bonds at a cutoff of 3.5 Å.
